# Clinical characteristics, prognosis, and fertility outcomes in patients with simple and complex endometrial hyperplasia: a comparative analysis

**DOI:** 10.1007/s00404-026-08356-9

**Published:** 2026-02-11

**Authors:** Jiayu Wei, Hong Wang, Haiyun Wang, Yingmei Wang, Wenyan Tian, Huiying Zhang

**Affiliations:** 1 Department of Gynecology and Obstetrics, Tianjin Medical University General Hospital, NO 154, Anshan Road, He Ping District, 300052 Tianjin, People’s Republic of China; 2 Tianjin Key Laboratory of Female Reproductive Health and Eugenics, 300052 Tianjin, People’s Republic of China; 3 Department of Gynecology and Obstetrics, Harbin Red Cross Central Hospital, Harbin, 150078 Heilongjiang People’s Republic of China

**Keywords:** Endometrial hyperplasia, Obesity, Prognosis, Fertility, LNG-IUS

## Abstract

**Objective:**

To analyze the difference in general clinical data, clinical manifestations, hysteroscopic manifestations, prognosis and fertility between patients with complex endometrial hyperplasia (CH) and simple endometrial hyperplasia (SH).

**Material and methods:**

Collected the medical records of 616 premenopausal endometrial hyperplasia (EH) patients from January 2012 to October 2023, of which 419 SH patients and 197 CH patients were included in the study. All the patients were followed up at least 12 months, and asked about the follow-up treatment plan, review, pregnancy and reproductive outcome of the patients with reproductive needs.

**Results:**

Obesity (P = 0.044), having diabetes or insulin resistance (P = 0.032) and polycystic ovary syndrome (PCOS) (*P* < 0.001) are risk factors for the occurrence of CH, while gravidity ≥ 1 (*P* = 0.045) is a protective factor for the occurrence of CH. Compared with the SH group, the reversal rate in the CH group was significantly lower (69.7% vs 83.6%, *P* < 0.001), while the rate of persistence, progression, recurrence and canceration were higher (*P* < 0.001). Compared with no treatment, oral progesterone (*P* < 0.001) and levonorgestrel-releasing intrauterine system (LNG-IUS) treatment (P < 0.001) could improve the prognosis of patients with EH. The live birth rate of the CH group was obviously lower than that of the SH group ( 42.3% VS 61.1%, *P* = 0.038). CH (OR = 2.68, 95%CI 1.12–6.39, *P* = 0.043) is an independent risk factor affecting the live birth rate of patients with EH.

**Conclusion:**

Obesity, diabetes, insulin resistance, PCOS and nulligravidity are risk factors for patients with EH, while gravidity ≥ 1 served as a protective factor, particularly against CH. The type of hyperplasia is associated with a low live birth rate. The prognosis of EH patients is usually poor, with a low reversal rate and a long reversal time. However, LNG-IUS can improve their prognosis.

**Supplementary Information:**

The online version contains supplementary material available at 10.1007/s00404-026-08356-9.

## What does this study add ?

**Table:** 

Our study identified obesity, diabetes/insulin resistance, and PCOS as independent risk factors for complex hyperplasia (CH), while gravidity ≥1 was protective factor. We added distinct hysteroscopic features in CH and early visual diagnosis. CH reduced live birth rates by 31% despite similar pregnancy rates, highlighting its impact on reproductive success. Our study included a large cohort and long follow-up over 12 months. Proposes long-term metabolic management (obesity/diabetes control) alongside LNG-IUS therapy to improve prognosis and prevent progressing to endometrial cancer.	

## Introduction

Endometrial hyperplasia (EH) is a non-physiological, non-invasive proliferative condition that results in an increase in endometrial volume. It is primarily characterized by changes in glandular structure (size and shape) and an elevated ratio of endometrial glands to stroma compared to normal endometrium [1]. According to the 2014 and 2020 WHO classification, EH is classified as endometrial without atypical hyperplasia by integrating the international endometrial intraepithelial neoplasia (EIN) classification. Simple endometrial hyperplasia (SH) and complex endometrial hyperplasia (CH) were classified as EH and atypical hyperplasia as atypical endometrial hyperplasia (AH) or EIN according to whether they were accompanied by cell atypia [2].

EH carries a 1% to 3% risk of progressing to endometrial cancer (EC), with a 75% to 100% response rate, while AH or EIN has a 25% to 33% risk of developing into EC [3]. Due to its strong association with EC, patients with EH should be closely monitored in clinical practice. A meta-analysis of 12 studies conducted by Travaglino in 2019 included a total of 804 patients without AH. Among them, 238 had SH and 566 had CH. The results indicated that the risk of SH progressing to EC was 2%, whereas the risk for CH was 12.4% (OR = 6.03, *P* < 0.001) [4]. This suggests that different pathological types carry varying risks of progressing to EC. Early identification and treatment of EH play a crucial role in preventing the development of EC. There are few studies related to the reproductive outcomes of patients with EH. Therefore, this study analyzed the clinical manifestations and prognosis of patients with SH and CH, aiming to explore the difference between the two groups of patients and provide evidence for the management and treatment of clinical patients with EH.

## Material and methods

### Patients

We included participants (1) whose pathological diagnoses were EH without atypia or AH; (2) who had undergone hysteroscopy and histopathological examination at Tianjin Medical University General Hospital; (3)premenopausal women.We excluded patients (1) who had hepatic or renal insufficiency; (2) who had EH without atypia or AH along with other malignant tumors; (3) who had a pregnancy and suspected pregnancy; (4) whose clinical and pathological data were missing; and (5) who had history of venous thrombosis or family history of deep vein thrombosis. Based on the inclusion and exclusion criteria, 616 patients were finally included from January 2012 to October 2023.

### Demographic and clinical characteristics

Demographic and clinical characteristics including patient age, age at menarche, body mass index (BMI), parity, gravidity, chief complaint, history of hypertension, type II diabetes, PCOS, degree of anemia, symptoms of abnormal uterine bleeding (AUB). The hysteroscopic features of patients diagnosed by hysteroscopy, dilatation and curettage were recorded (including endometrial thickness, color, blood vessels, tissue status, whether accompanied by polyps and so on). The patients were grouped according to SH and CH.

### Follow up

Follow-up was performed every 3–6 months for a mean duration of 57 months. Patients' weight, BMI and symptoms of AUB, method of conception and outcome of fertility in patients with fertility need and endometrial pathology were recorded at each routine visit. All patients were followed up until May 31 2024 to compare the rate of improvement of finalAUB symptoms, the rate of improvement of ultrasound endometrial imaging, and the rate of remission and recurrence in each group.

### Pathological diagnoses

Diagnostic evaluation by endometrial sampling by hysteroscopy or curettage was performed on all patients in this study. Gynecological pathologists from the Pathology Department of Tianjin Medical University General Hospital made the pathological diagnoses. The final study diagnosis was the most severe pathological diagnosis. Pathological diagnoses were made according to the International Federation of Gynaecological Pathology (FIGO) grading criteria.

### Statistical analyses

Statistical analyses were conducted using SPSS version 26.0. Normality of continuous variables was assessed using the Kolmogorov–Smirnov test. Data that followed a normal distribution are presented as mean ± standard deviation (SD), while non-normally distributed variables are expressed as median (interquartile range). Between-group comparisons for continuous variables were performed using the independent-samples t-test or Mann–Whitney U test as appropriate. Categorical variables are presented as frequencies and percentages, with comparisons made using the chi-square test or Fisher’s exact test. A P-value < 0.05 was considered statistically significant. Multivariate logistic regression analysis was performed on clinically relevant or statistically significant variables identified in univariate analysis. An enter method was used for variable selection, where all candidate variables were included in the model regardless of their individual statistical significance.

## Results

### Patients’ characteristics

The CH group was significantly younger than the SH group, with a mean age of 36.99 ± 7.62 years compared to 40.47 ± 7.88 years (*P* < 0.001). The CH group also had a higher average BMI (27.82 ± 5.32 kg/m^2^ vs. 25.78 ± 4.28 kg/m^2^, *P* < 0.001) and a greater prevalence of obesity (43.1% vs. 27.0%, *P* < 0.001). Reproductive history differed significantly, with lower proportions of patients in the CH group having at least one gravidity (46.2% vs. 70.9%, *P* < 0.001) or at least one parity (50.9% vs. 70.9%, *P* < 0.001). Metabolic and endocrine profiles further distinguished the three groups. The CH group showed a higher rate of diabetes or insulin resistance (32.5% vs. 15.5%, *P* < 0.001) and a greater prevalence of PCOS (31.5% vs. 9.5%, *P* < 0.001). No significant difference was observed in the prevalence of hypertension (*P* = 0.072). Patients with CH exhibited a significantly longer mean medical history compared to those with SH (20 months vs 6 months, *P* < 0.001). (Supporting Information Table S1).

Key variables from the univariate analysis were included in the multivariate logistic regression model. The results indicated that obesity (OR = 1.49, 95% CI 1.01–2.21, *P* = 0.044), diabetes or insulin resistance (OR = 1.64, 95% CI 1.04–2.57, *P* = 0.032), and PCOS (OR = 2.74, 95% CI 1.64–4.60, *P* < 0.001) were identified as independent risk factors for CH. Gravidity ≥ 1 was found to be a protective factor for CH (Supporting Information Table S2).

### Analysis of EH patients’ clinical manifestations

Premenopausal patients with EH were evaluated based on their primary complaints. Clinical presentations were categorized into the following six types: asymptomatic, intermenstrual bleeding, prolonged menstrual period, oligomenorrhea, irregular menstruation, and changes in menstrual volume. Among these, changes in menstrual volume and irregular menstruation showed statistically significant differences between groups (*p* < 0.001) (Supporting Information Figure S3).

### Hysteroscopic manifestations of EH patients

A total of 546 patients were diagnosed with EH by hysteroscopy, including 379 with SH and 167 with CH. Hysteroscopic images were evaluated based on endometrial color (normal: pink; abnormal: gray, yellow, white, or diffusely congested), endometrial thickness (thin, medium, unevenly thickened, or uniformly thickened), presence of abnormal vessels, and endometrial status (fresh or brittle). Polyps were also noted. Compared to SH patients, those with CH showed a higher incidence of abnormal endometrial color (χ^2^ = 16.064, *P* < 0.001), uneven endometrial thickening (χ^2^ = 15.520, *P* = 0.001), abnormal vessels (χ^2^ = 52.195, *P* < 0.001), and brittle tissue (χ^2^ = 5.451, *P* = 0.020). These hysteroscopic features can help clinicians identify suspicious areas and guide targeted biopsy to improve diagnostic accuracy and ensure appropriate management (Supporting Information Figure S4, Table S5).

### Prognosis analysis of EH patients

The reversion time is the interval between two hysteroscopies with normal pathology. The reversal rate of the CH group within 12 months was lower than that of the SH group (69.7% vs. 83.6%, *P* < 0.001). At follow-up, the prognosis of patients was categorized into four outcomes: reversion, persistence, progression, and recurrence. In the cohort of CH patients, the reversal rate was significantly lower compared to SH patients (70.6% vs. 90.7%, *P* < 0.001). Additionally, the persistence rate was significantly higher in CH patients than that in SH patients (18.1% vs. 3.8%, *P* < 0.001), as was the progression rate (7.7% vs. 4.3%, *P* < 0.001) and recurrence rate (3.6% vs. 1.1%, *P* < 0.001) (Supporting Information Figure S6).

Patients were stratified by treatment into untreated, oral progesterone, and LNG-IUS groups. Reversal rates were analyzed separately for SH and CH patients. Among SH patients, the reversal rate was highest in the LNG-IUS group (90.6%), followed by the oral progesterone group (87.0%), and lowest in the untreated group (52.5%), with a statistically significant difference among the three groups (χ^2^ = 17.557, *P* < 0.001). For CH patients, the reversal rate was 79.8% in the oral progesterone group, 77.3% in the LNG-IUS group, and 21.9% in the untreated group, which also represented a significant difference (χ^2^ = 41.669, *P* < 0.001). Multivariate logistic regression analysis identified CH (OR = 3.97, 95%CI 2.55–6.18, *P* < 0.001), older age (OR = 1.04, 95%CI 1.01–1.07, *P* = 0.016), and nulligravidity (OR = 2.34, 95%CI 1.08–5.06, *P* = 0.031) as risk factors for poor prognosis in EH patients. Compared to untreated, oral progesterone (OR = 0.25, 95%CI 0.16–0.41, P < 0.001) and LNG-IUS (OR = 0.22, 95%CI 0.11–0.45, *P* < 0.001) improved the prognosis of EH patients(Supporting Information Figure S7).

In the follow-up results, The canceration rate of patients with CH was higher than that of patients with SH (3% vs 0.7%, P < 0.001). The hysterectomy rate of the CH group was significantly higher than that of the SH group (16.4% vs 6.7%, 2 = 14.047, *P* < 0.001). Both are statistically significant (Supporting Information Table S8).

A total of 161 patients with preserved fertility, clear fertility demands, and completed treatment were included in the study, and there were 71 patients with CH and 90 patients with SH. The pregnancy rate in CH patients was lower than that in SH patients(73.2% vs 75.6%), but there was no significant statistical difference (χ^2^ = 0.112, P = 0.738).However, the live birth rate was significantly lower in CH patients(42.3% vs 61.1%, χ^2^ = 3.599, P = 0.038). Multivariate analysis confirmed CH as an independent risk factor for reduced live birth (OR = 2.68, 95% CI: 1.12–6.39, *P* = 0.043) (Table S9).

## Discussion

Aging, early menarche or late menopause, infertility, obesity, diabetes or insulin resistance, PCOS, estrogen-secreting tumors, and long-term use of medications such as tamoxifen have all been identified as risk factors for EH [5]. Our study showed that Gravidity ≥ 1 is an essential protective factor for CH, which may be related to the protective effect of high progesterone levels during pregnancy on the endometrium. The main cause of EH is exposure to estrogen stimulation without progesterone antagonism [6]. Infertility, a known risk factor for EH, is also a significant clinical manifestation in women of reproductive age. In patients with PCOS, concurrent hyperinsulinemia, ovulatory dysfunction, hyperandrogenemia, and chronic low-grade inflammation are key factors contributing to the onset and progression of EH [7].

The main clinical manifestations of EH is AUB. In premenopausal women, it is manifested as changes in menstrual frequency, regularity, menstrual volume, intermenstrual bleeding, and prolonged menstrual period. It is estimated that endometrial lesions (hyperplasia and carcinoma) account for 10%–20% of postmenopausal bleeding cases [8]. Ianier et al. [9]established a hysteroscopic scoring system based on 435 endometrial biopsies, reporting a sensitivity and specificity of 48.7% and 85.2% for non-atypical endometrial hyperplasia, 63.3% and 90.4% for AH, and 95.4% and 98.2% for EC, respectively. In our study, hysteroscopy showed that the endometrium of the CH was gray, the endometrial thickness was unevenly thickened, the proportion of irregular blood vessels increased, and the tissue was more brittle. There was no statistically significant difference in the presence or absence of endometrial polyps between the SH and CH patients. Therefore, it suggests that when observing endometrial polypoid hyperplasia under hysteroscopy, we should conduct a targeted biopsy based on the conditions of other endometrium outside the polyps. Relying solely on polypectomy may increase the risk of missing underlying hyperplastic lesions.

The results demonstrated a higher prevalence of obesity and overweight individuals in the CH group, suggesting that obesity plays a significant role in the progression of EH. This is consistent with the findings of Wise et al. [10]. The study by Campagnoli [11] showed that the risk of EH was increased by 4 times for those with BMI > 30 kg/m2. Hale et al. [12] found that increased conversion of androstendione to estrone by tissue aromatase, increased free estrogen due to decreased sex hormone-binding globulin, increased insulin-like growth factor, and decreased progesterone secondary to ovulation disorder relative to baseline are mechanisms of increased risk of obesity-associated endometrial lesions. Excess adipose tissue also leads to a decrease in chronic inflammatory and anti-inflammatory cytokines secondary to the production of pro-inflammatory adipokines. Inflammation leads to a decrease in tumor necrosis factor-α (TNF-α), proliferating cell nuclear antigen (PCNA), and epithelial growth factor (EGF) mRNA. It also increases the production of Fas mRNA and IGF-1 receptor (IGF-1R), affecting endometrial DNA repair [13].

Diabetes or insulin resistance also plays an essential role in the occurrence and progression of EH. Holm et al. 's [14] research results showed that the risk of EH leading to EC in diabetic women was 2 times and 4 times higher than that of non-diabetic women with BMI and age, respectively. Endometrium is the target of insulin, and insulin inhibits endometrial decidualization. Insulin also stimulates the production of androgens in the ovaries and adrenal glands, and acts as a endometrial mitogen by increasing the action of insulin growth factor on the endometrium. Zhou Xueyan et al. [15] suggested that T2DM can promote the progression of EH by regulating GalNT2-mediated EGFR phosphorylation and enhancing cell proliferation.

At present, the main treatment methods for EH are progesterone therapy and hysterectomy. For patients without AH, progesterone therapy has become the first choice. Progesterone can inhibit EH by antagonizing the effect of estrogen, reducing adenocytosis by inducing cell apoptosis, and inhibiting muscle angiogenesis below the lesion of EH [16]. At present, the commonly used for therapy are oral progesterone, LNG-IUS, etc. The results showed that LNG-IUS had a better therapeutic effect on CH and SH. The reversal rate was higher, followed by oral progesterone, which was consistent with previous findings [17–19]. LNG-IUS can directly act on local endometrial lesions and reduce adverse reactions of whole body progesterone [20]. In a study by Shen et al. [21]involving patients with non-atypical endometrial hyperplasia, treatment with LNG-IUS was associated with a higher regression rate, shorter time to regression, lower incidence of drug resistance, and a reduced hysterectomy rate compared to oral progesterone therapy. This further proves the effectiveness and safety of LNG-IUS in EH.

Widra et al. [22] reported that the risk of progression to EC was about 1% for SH and 3% for CH, respectively. Bernstein et al. showed that 2% of CH cases (8/390) progressed to EC  [23], and our findings are similar. Although the risk of progressing to EC is low, it warrants attention. PALOMBA S et al. results show [24] that the endometrial dysfunction, decreased receptivity, changes in the immune environment, chronic inflammatory state, damage to the decidualization of endometrial cells, and the influence of their own complications in patients with EH can affect the implantation of fertilized eggs and embryonic development, and the risk of pregnance-related complications also increases accordingly. Our study highlights a critical clinical distinction in reproductive outcomes between endometrial hyperplasia subtypes. Although pregnancy rates were comparable, CH patients exhibited a significantly lower live birth rate than SH patients, establishing CH as an independent risk factor for reduced live birth. These findings suggest that the primary challenge for CH patients is not achieving conception, but rather maintaining the pregnancy to full term.

Clinicians should clearly communicate the elevated risk of pregnancy loss to CH patients seeking fertility, helping to establish realistic expectations early in the treatment process. A proactive and multidisciplinary approach is recommended. Following endometrial histological regression, timely referral to assisted reproductive technology (ART) may enhance endometrial receptivity and reduce the risk of disease recurrence [25]. Moreover, due to the compromised endometrial environment and frequent co-occurrence of metabolic disorders, pregnancies in CH patients should be considered high-risk and managed in collaboration with maternal–fetal medicine specialists to optimize monitoring, mitigate complications, and improve the likelihood of a successful term pregnancy. This study has several limitations. Its retrospective design and single-center setting may introduce selection bias and limit the generalizability of the results. The follow-up duration, although substantial, might be insufficient to evaluate long-term outcomes such as recurrence or cancer progression. We recognize that interobserver variability in hysteroscopic interpretation and pathological assessment may affect diagnostic accuracy. Variations in clinician experience and expertise could introduce bias, influencing the results. The fertility analysis was restricted to a self-selected subgroup of treatment-completed patients with fertility intentions, which may not represent the broader EH population. Finally, despite statistical adjustments, residual confounding from unmeasured variables cannot be excluded. Future investigations would benefit from prospective, multi-center designs with extended follow-up duration.

## Conclusion

During the treatment of patients with EH, we should pay attention to long-term management, control complications such as obesity and diabetes, and eliminate high-risk factors. It plays a crucial role in controlling the progression of EH and promoting prognosis. At present, internationally it is recommended that EH patients should actively engage in pregnancy after achieving complete remission, and assisted reproductive technology is recommended as the mode of pregnancy. Our findings further indicate that the live birth rate in CH patients is relatively low. This underscores the importance of closely monitoring the reproductive outcomes of CH patients during their treatment. Further research is required to investigate the subsequent pregnancy outcomes and potential pregnancy complications in this patient population.

## Supplementary Information

Below is the link to the electronic supplementary material.

Supplementary file1 (DOCX 1117 KB)

## Data Availability

The datasets generated and/or analysed during the current study are not publicly available due to privacy restraints but are available on request.

## Abbreviations

*EH*: Endometrial hyperplasia
*CH*: Complex endometrial hyperplasia
*SH*: Simple endometrial hyperplasia
*AH*: Atypical endometrial hyperplasia
*EIN*: Endometrial intraepithelial neoplasia
*PCOS*: Polycystic ovary syndrome
*AUB*: Abnormal uterine bleeding
