# Supplementary material for: Clinical characteristics, prognosis, and fertility outcomes in patients with simple and complex endometrial hyperplasia: a comparative analysis
Source: Arch Gynecol Obstet. 2026 Feb 11;313(1):87. doi: 10.1007/s00404-026-08356-9 (PMC12894132; doi:10.1007/s00404-026-08356-9)
Supplement: Supplementary file 1 — Supplementary file1 (DOCX 1117 KB) [file 404_2026_8356_MOESM1_ESM.docx]

Table and figure

Table S1：Clinical characteristics of CH group and SH group

|  | CH (n=197) | SH (n=419) | t/χ^2^ | P |
| --- | --- | --- | --- | --- |
| Age | 36.9 (7.62) | 40.47 (7.88) | 5.312 | ＜0.001 |
| BMI（kg/m^2^） | 27.82 (5.32) | 25.78 (4.28) | 4.723 | ＜0.001 |
| Obesity |  |  | 20.115 | ＜0.001 |
| Normal | 112 (56.9%) | 305 (72.8%) |  |  |
| Obesity | 85 (43.1%) | 113 (27.2%) |  |  |
| Gravidity≥1 | 108 (54.8%) | 330 (78.8%) | 37.367 | ＜0.001 |
| Parity≥1 | 91 (46.2%) | 297 (70.9%) | 35.038 | ＜0.001 |
| Hypertension | 22 (11.2%) | 70 (16.7%) | 3.236 | 0.072 |
| Diabetes/insulin resistance | 64 (32.5%) | 65 (15.5%) | 23.320 | ＜0.001 |
| PCOS | 62 (31.5%) | 40 (9.5%) | 46.623 | ＜0.001 |

***Note:* Data are presented as mean ± SD or n (%).**

Abbreviations: complex endometrial hyperplasia (CH), simple endometrial hyperplasia (SH), body mass index (BMI), polycystic ovary syndrome (PCOS)

Table S2：Prognostic factors of developing CH

|  | OR | CI 95% | P |
| --- | --- | --- | --- |
| Age | 1.00 | 0.97-1.03 | 0.948 |
| Obesity | 1.49 | 1.01-2.21 | 0.044 |
| Diabetes or insulin resistance | 1.64 | 1.04-2.57 | 0.032 |
| PCOS | 2.74 | 1.64-4.60 | ＜0.001 |
| Gravidity≥1 | 0.50 | 0.25-0.99 | 0.045 |
| Parity≥1 | 0.83 | 0.42-1.68 | 0.611 |

Figure S3: The proportion of clinical manifestations in patients with CH and SH


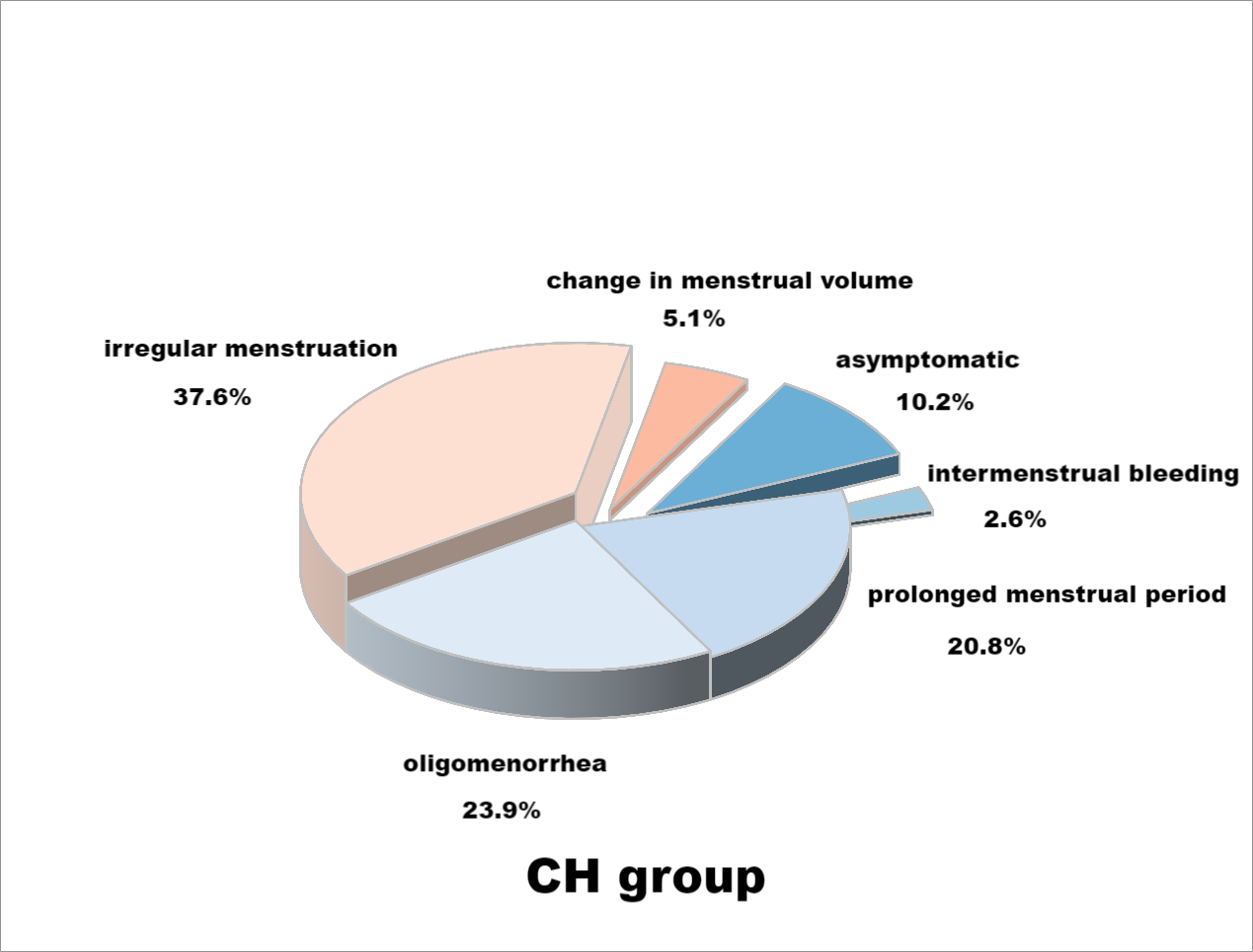


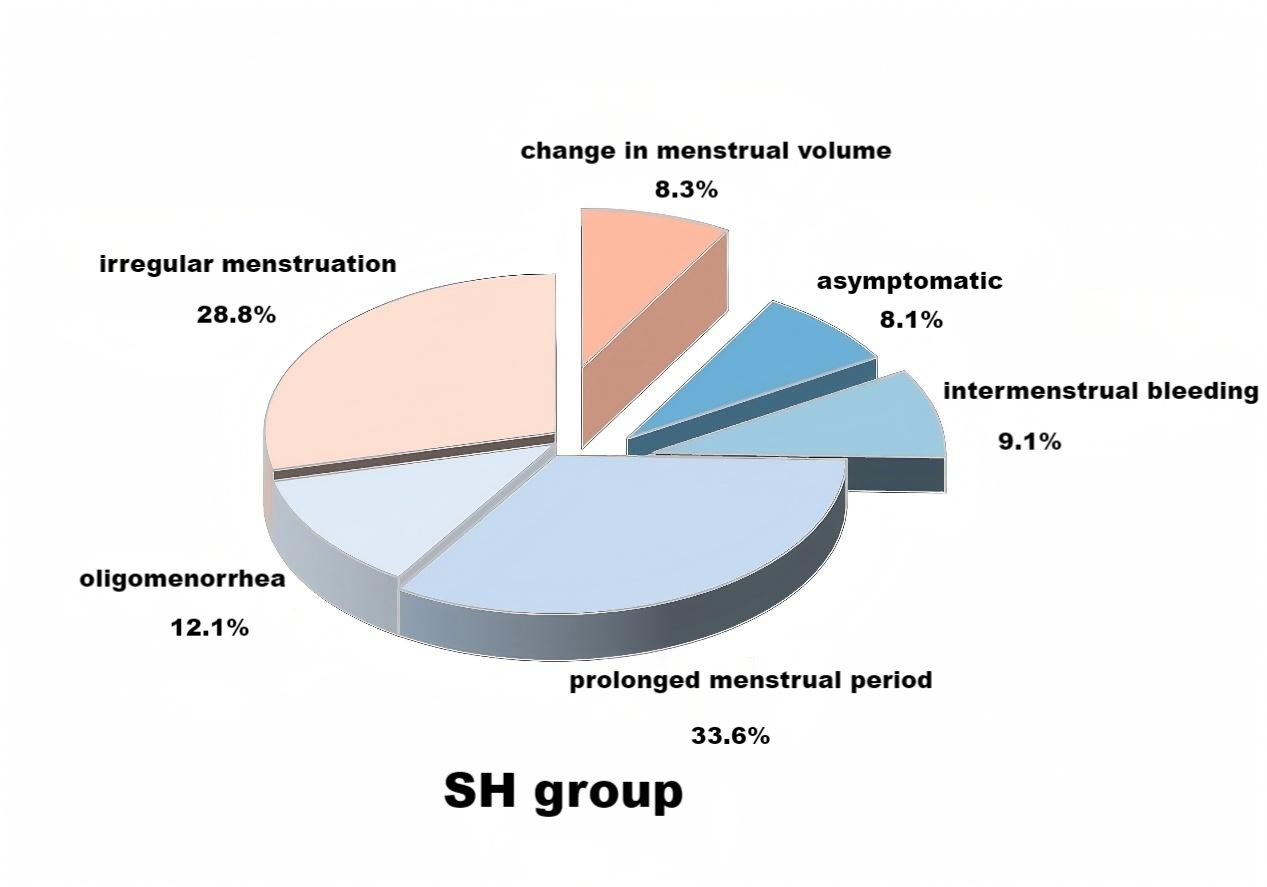


Figure S4: Representative hysteroscopy images of CH and SH patients: SH shows mild endometrial thickening with regular vasculature, while CH exhibits irregular, thickened endometrial tissue with abnormal vasculature and a more prominent, uneven appearance


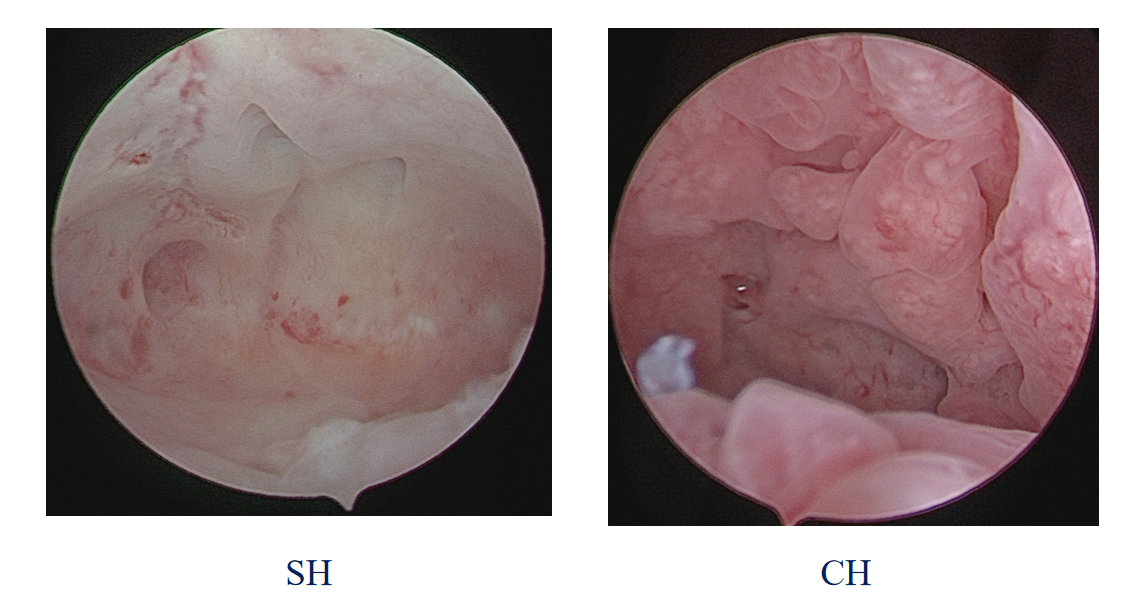


Table S5: Hysteroscopic manifestations of CH group and SH group

|  | CH (n=167) | SH (n=379) | χ^2^ | P |
| --- | --- | --- | --- | --- |
| Endometrial color |  |  | 16.064 | ＜0.001 |
| Abnormal | 66 (39.5%) | 87 (22.8%) |  |  |
| Normal | 101 (60.5%) | 284 (77.2%) |  |  |
| Endometrial  thickness |  |  | 15.520 | 0.001 |
| thin | 5 (3.0%) | 15 (4.0%) |  |  |
| medium thick | 37 (22.2%) | 128 (33.8%) |  |  |
| uniform | 54 (32.3%) | 136 (35.9%) |  |  |
| nonuniform | 71 (42.5%) | 100 (26.4%) |  |  |
| Irregular blood vessel |  |  | 52.195 | ＜0.001 |
| yes | 126 (75.4%) | 160 (42.3%) |  |  |
| no | 41 (24.6%) | 219 (57.7%) |  |  |
| Endometrial status |  |  | 5.451 | 0.020 |
| fresh | 110 (65.9%) | 290 (75.5%) |  |  |
| rittle | 57 (34.1%) | 89 (24.5%) |  |  |
| Endometrial polyps |  |  | 2.280 | 0.131 |
| yes | 101 (60.2%) | 203 (53.5%) |  |  |
| no | 66 (39.8%) | 176 (46.5%) |  |  |

Figure S6: Follow-up of CH group and SH group.

Figure S7:  Treatment efficacy of LNG-IUS and oral progesterone on reversion rates in EH compared to untreated controls.


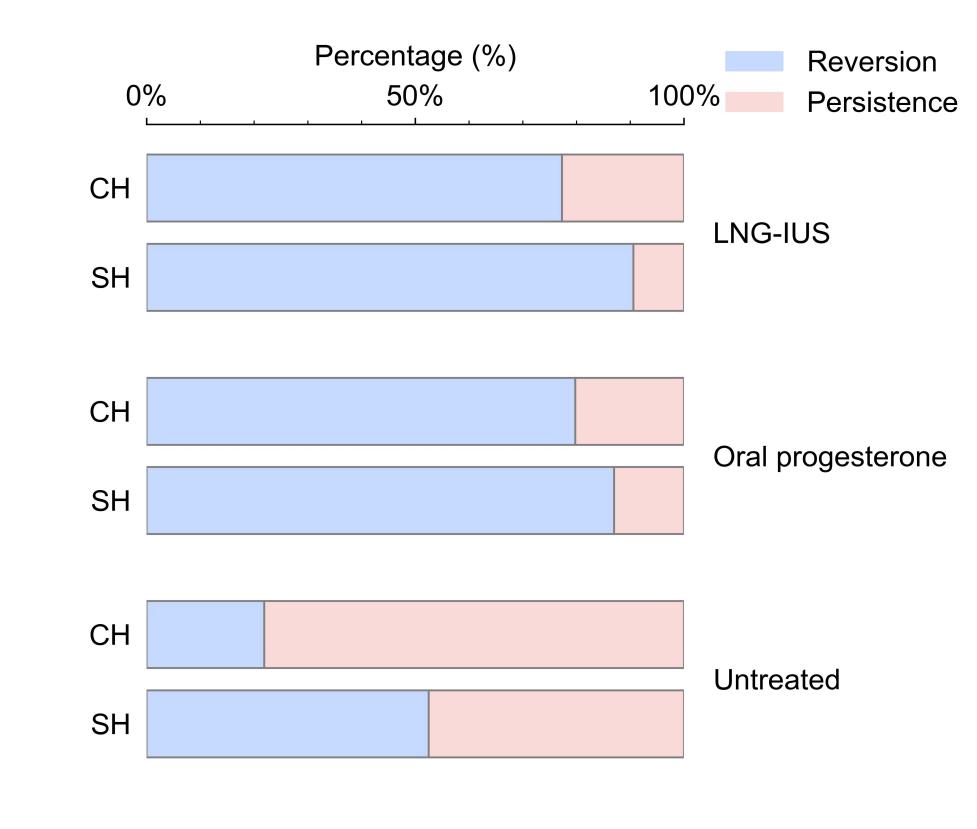


Abbreviations: levonorgestrel-releasing intrauterine system (LNG-IUS)

Table S8: Prognostic factors of EH analyzed by multivariate logistic regression

|  | OR | CI 95% | P |
| --- | --- | --- | --- |
| CH | 3.97 | 2.55-6.18 | ＜0.001 |
| Obesity | 1.00 | 0.63-1.60 | 0.987 |
| Age | 1.04 | 1.00-1.07 | 0.016 |
| Gradivity=0 | 2.34 | 1.08-5.06 | 0.031 |
| Parity=0 | 0.53 | 0.25-1.09 | 0.081 |
| PCOS | 1.62 | 0.77-3.39 | 0.202 |
| Therapy |  |  |  |
| Untreated |  |  |  |
| Progesterone | 0.25 | 0.16-0.41 | ＜0.001 |
| LNG-IUS | 0.22 | 0.11-0.45 | ＜0.001 |

Table S9: Comparison of pregnancy rate and live birth rate between the CH group and the SH group.

|  | CH (n=71) | SH (n=90) | χ^2^ | P |
| --- | --- | --- | --- | --- |
| Pregnancy rate | 73.2%  (52/71) | 75.6%  (68/90) | 0.112 | 0.738 |
| Live birth rate | 42.3%  (30/71) | 61.1%  (55/90) | 3.599 | 0.038 |
